# Observation of transition from superfluorescence to polariton condensation in CsPbBr_3_ quantum dots film

**DOI:** 10.1038/s41377-024-01378-5

**Published:** 2024-01-30

**Authors:** Danqun Mao, Linqi Chen, Zheng Sun, Min Zhang, Zhe-Yu Shi, Yongsheng Hu, Long Zhang, Jian Wu, Hongxing Dong, Wei Xie, Hongxing Xu

**Affiliations:** 1 State Key Laboratory of Precision Spectroscopy, East China Normal University, Shanghai, 200241 China; 2 Key Laboratory of Materials for High-Power Laser, Shanghai Institute of Optics and Fine Mechanics, Chinese Academy of Sciences, 201800 Shanghai, China; 3 Collaborative Innovation Center of Extreme Optics, Shanxi University, Taiyuan, Shanxi 030006 China; 4 Chongqing Key Laboratory of Precision Optics, Chongqing Institute of East China Normal University, Chongqing, 401121 China; 5 CAS Center for Excellence in Ultra-intense Laser Science, Shanghai, 201800 China; 6 School of Physics and Technology, Center for Nanoscience and Nanotechnology, Wuhan University, Wuhan, 430072 China

**Keywords:** Polaritons, Polaritons

## Abstract

The superfluorescence effect has received extensive attention due to the many-body physics of quantum correlation in dipole gas and the optical applications of ultrafast bright radiation field based on the cooperative quantum state. Here, we demonstrate not only to observe the superfluorescence effect but also to control the cooperative state of the excitons ensemble by externally applying a regulatory dimension of coupling light fields. A new quasi-particle called cooperative exciton-polariton is revealed in a light-matter hybrid structure of a perovskite quantum dot thin film spin-coated on a Distributed Bragg Reflector. Above the nonlinear threshold, polaritonic condensation occurs at a nonzero momentum state on the lower polariton branch owning to the vital role of the synchronized excitons. The phase transition from superfluorescence to polariton condensation exhibits typical signatures of a decrease of the linewidth, an increase of the macroscopic coherence as well as an accelerated radiation decay rate. These findings are promising for opening new potential applications for super-brightness and unconventional coherent light sources and could enable the exploitation of cooperative effects for quantum optics.

## Abstract (web summary)

We not only confirmed the superfluorescence effect, but also demonstrated the phase transition to cooperative exciton-polariton condensation. This was achieved by applying a regulatory dimension of light field.

## Graphical abstract

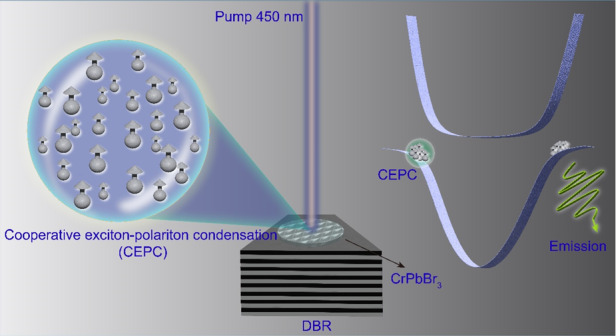

## Introduction

Superfluorescence (SF)^1–3^, as a cooperative radiation effect originating from vacuum quantum fluctuations, is an ideal platform for studying many-body correlation mechanisms in excitons ensemble and for developing optically ultrafast techniques on bright quantum light sources^4–6^. Since the short, intense emission bursts were first observed in colloidal semiconductor nanocrystals such as cesium lead halide (CsPbX_3_, *X* = Cl, Br) perovskite quantum dots (QDs)^7–12^, exploring the SF effect over various materials and different operating temperatures has received extensive attention. Recently, a research group first revealed the quantum phase transition in a hybrid perovskite system at room temperature^11^. The underlying mechanism of the robust protection of the coherent macroscopic state in the material is still under debate. However, current works mainly focus on studying and discussing the establishment of the SF itself. Here, we report the way to control the radiation behavior of cooperative excitons in SF systems by applying a new regulatory dimension of coupling light field.

In fact, extensive research has been dedicated to investigating the interactions between perovskite single crystals and DBRs^13–16^ over the years. There have also been concerted efforts to develop low-threshold lasers through the integration of quantum dots with DBRs^17–21^. However, here we focus on elucidating the formalism of a novel class of quasiparticles known as cooperative exciton-polaritons (CEPs), which emerge due to the strong coupling between cooperative excitons and optical modes. Unlike the traditional generation of the exciton-polaritons^22–26^ in semiconductor microcavities coupled by individual excitons and light filed, CEPs behave very differently for the sake of the many-body correlation built up among the individual excitons. On the one hand, the involved correlated excitons can enhance the Rabi splitting by a factor of $\sqrt{N}$ (*N* is the number of cooperative excitons)^27–29^. Thus, it allows the strong coupling to take place beyond what is possible at the individual dipole level (see supplementary information). On the other hand, due to the additional regulatory dimension in the excitons ensemble and the small effective mass of CEPs, one could study the phase transition from SF to cooperative exciton-polariton condensation (CEPC) from the cryogenic temperature up to room temperature. The dual light-matter nature of the CEPC facilitates its potential applications in quantum simulation^30–32^, untraditional coherent light sources^33–36^ and all-optical polarization logic devices^37–39^. Though the exciton-polariton has been well achieved and studied in various systems^22,40–42^, the strong coupling of cooperative excitons and light fields has yet to be reported, let alone to approach the phase transition.

Here, we demonstrate the above proposal in a light-matter hybrid structure of a thin film stacked by perovskite QDs and a Distributed Bragg Reflector (DBR). The CEPs are formalized by coupling an ensemble of superfluorescent excitons to a selected Bragg mode^43,44^. When the excitation power is above the density threshold, polariton condensate appears at a nonzero momentum state on the lower branch of CEP dispersions. We find that the system below the CEPC transition threshold may be well described by a theoretical model like the two-channel model often used in the conventional cavity exciton-polariton systems (see methods). Numerical calculations based on this model reveal that the strong exciton-Bragg mode coupling alters the total density of states of the system, and the energy-momentum point with the maximum density of states exactly corresponds to the CEP condensate point above the threshold. Our work may stimulate ultrafast control techniques of many-body quantum states^45,46^ and the high-quality nano-/micro-devices on quantum computing and on-chip lasers^47,48^.

## Results

**Fig. 1:**
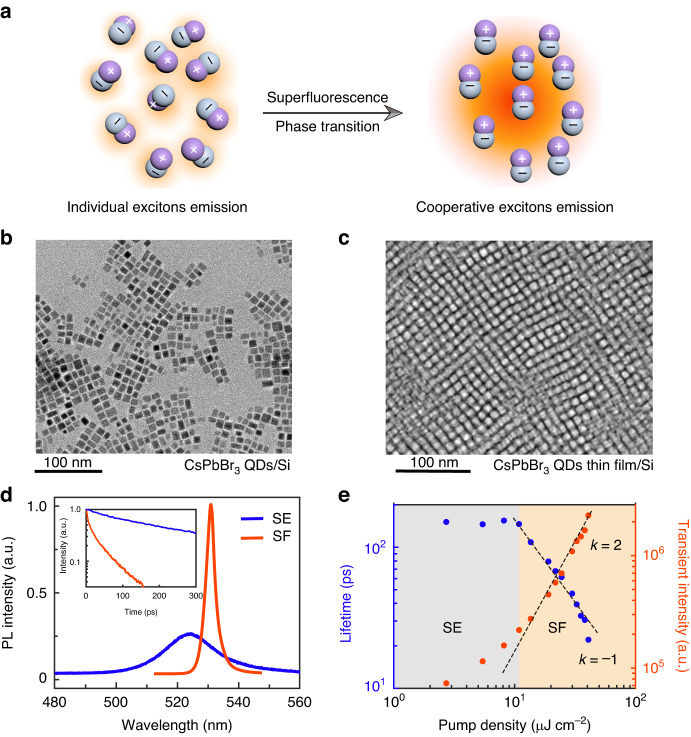
**Superfluorescence effect of excitons in a CsPbBr**_**3**_ **QDs thin film on silicon**. **a** Schematic of the build-up process of superfluorescence (SF). Individual excitons exhibit random phases (the dipole moment consists of the hole and the electron and are labeled as ‘+’ and ‘−’). After phase transition, the phases of excitons are synchronized (aligned in the same orientation) and form a giant dipole moment. **b**, **c** TEM images of unassembled monodispersed and closely packing CsPbBr_3_ QDs, respectively. The typical thickness of the thin film CsPbBr_3_ ranges from 200 nm to 500 nm. **d** Time-integrated photoluminescence spectra for spontaneous emission (SE, blue line) and SF (red line), respectively. The typical peak wavelengths for SE and SF are 525 nm and 530 nm, respectively. The inset shows the time-resolved spectra of SE (blue line) and SF (red line). The radiation lifetimes are extracted as 150 ps and 22 ps. **e** Pump density vs. the transient peak intensity and the radiation lifetime plot in log-log scale. The phase domain can be determined by the black dashed fitting lines for the trends *y* = x^k^. The gray area represents the SE interval, and the orange area represents the SF interval. The typical k values in the SF interval are 2 for the transient radiation intensity and −1 for the lifetime

An ensemble of excitons can behave very differently if they interact coherently with an external light field which can result in a phase transition from the spontaneous emission (SE) to a many-body quantum phenomenon called superflurescence (SF) (shown in Fig. 1a). Here we prepare two different samples: one is the high-quality CsPbBr_3_ QDs thin film (shown in Fig. 1c, see also Methods), and the other is the unassembled monodispersed CsPbBr_3_ QDs illustrated in Fig. 1b as the control sample. The thin film consists of closely packing QDs with the typical thickness in a range from 200 nm to 500 nm. The photoluminescence (PL) measurements are performed at 10 K under the same proper excitation pump power on both samples and are displayed in Fig. 1d. It shows typical signatures of the intensity enhancement and wavelength redshift from the spontaneous emission (525 nm) of individual excitons (control sample) to the collective emission (530 nm) of cooperative excitons (QDs thin film), which is consistent with the previously reported self-organized CsPbBr_3_ quantum dot superlattice^10^. Burnham–Chiao ring effect and the increased time-correlated coherence (see supplementary information) can further help to indicate that our sample has approached the superfluorescence phase. In addition, due to the higher rate of absorbing and releasing energy in a correlated state, the shorter radiation decay time in the SF is expected and is determined by time-resolved PL measurements. The radiation lifetimes are calculated by fitting with an exponential decay function, and they are 150 ps and 22 ps for SE and SF separately. Later, we carry out the pump density dependence measurements over the QDs thin film. The excitation density is plotted with regard to the transient peak intensity and the radiation lifetimes. We find that below the threshold, the individual constituent of the ensemble possesses no correlations with each other and shows a random arrangement of the dipole moments. When above the threshold, the excitons are strongly affected by the disturbance of the vacuum field, thereafter the coherence starts to build up, thus the excitons behave in the same manner and resulting in superradiant emission. Based on it, we can categorize the phase domains into SE and SF as shown in Fig. 1e. In the SF interval, the measured values are fit to *y* = x^k^. The best fit of the k values is 2 for the transient peak intensity and −1 for the lifetimes, representing the feature values for the excitons that stay in the correlated state^1,3,10,11^. It should be noted that the rest of our work is mainly concentrated on the SF interval and beyond (CEPC) in the QDs thin film sample.

**Fig. 2:**
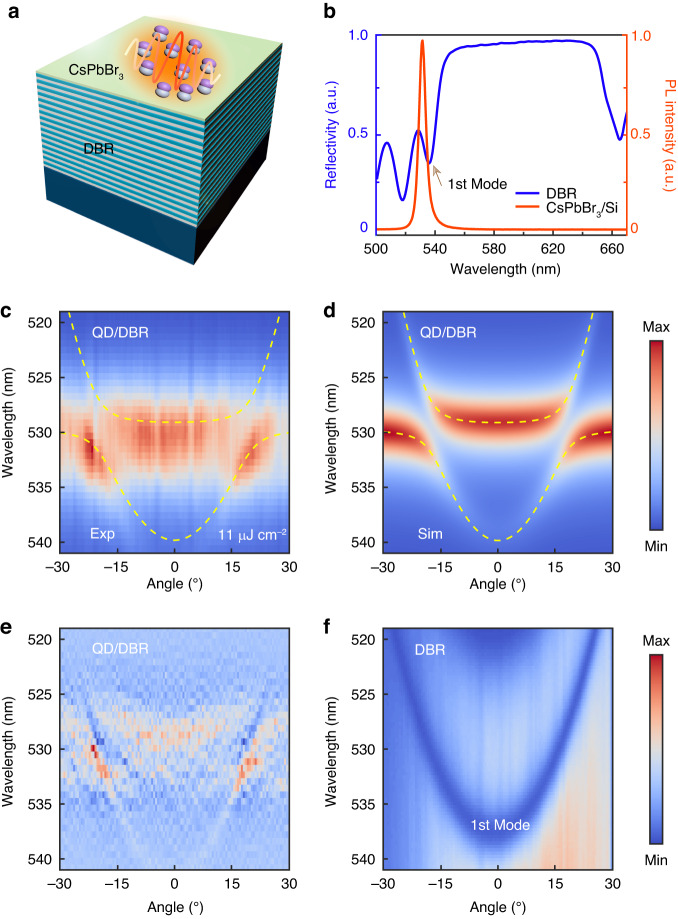
**Strong coupling between cooperative excitons and the first-order Bragg mode**. **a** Schematic diagram of the full sample structure, where on the topmost layer is the CsPbBr_3_ QDs thin film, and beneath it is a 16 pairs DBR grow on a silicon substrate. **b** SF emission of QDs thin film on silicon and reflectivity spectrum of the bare DBR measured at 10 K. **c** Angle-resolved measured PL spectra for the QDs thin film on DBR. The band of the cooperative excitons is split up by coupling to the first Bragg mode. The observed Rabi splitting energy is ~ 21.6 meV. **d** Calculated polaritonic density of states as a function of angle and wavelength. The yellow dashed lines in (**c**, **d**) are tracing the dispersion of the polariton modes. **e** The second-order derivative diagram for (**c**). **f** Angle-resolved reflectivity spectra for the bare DBR, the 1^st^ Bragg mode is shown at 537 nm

Figure 2a illustrates the schematic of the full structure design in the order of Si/DBR/CsPbBr_3_ QDs thin film from the bottom up. A similar sample made of the unassembled monodispersed QDs is prepared for control. We grow a sixteen-pairs (SiO_2_/SiN_x_) Distributed Bragg Reflector (DBR) by plasma-enhanced chemical vapor deposition (PECVD) with the typical first-order Bragg mode at the shorter wavelength side demonstrated by the reflectivity spectrum, as shown in Fig. 2b. The highly correlated excitons’ superradiant emission is at 530 nm and the designed first-order Bragg mode is at 537 nm with a Q factor of 300. Thus, it indicates the as-grown sample is −26 meV detuned.

The CsPbBr_3_ QDs thin film is then prepared on top of the DBR the same way as we do on the silicon substrate. A pulse laser with a pump wavelength of 450 nm and a repetition frequency of 200 kHz is employed to perform the angle-resolved PL measurements to investigate the modification of emission from the coupled hybrid states. It is worth noting that the variation in thin film thickness may impact the formation of the CEPC, however, the size of the tested spot is significantly smaller than the range of thickness fluctuations in the region. Consequently, we observed a uniform area under the pump laser, and the data presented here was acquired from a region with a thickness of 200 nm. (see also Methods). The angle has a one-to-one correspondence with in-plane momentum (${k}_{\parallel }$) through ${k}_{\parallel }=(\omega /c)(\sin \theta )$, c is the speed of light. Figure 2c presents the energy band gap of the cooperative excitons that appears at nearly 17° where the Bragg mode goes through. The typical strong coupling characteristics can be observed experimentally. The part of the energy band at the smaller angles is lifted while the rest part of the band at the larger angles is depressed down. Therefore, it indicates that the system may enter a strong coupling regime. To further confirm it, we calculate the excitonic density of states of the cooperative exciton-Bragg modes coupling system (Fig. 2d) and compare it with the angle-resolved PL spectra in Fig. 2c. The dashed yellow lines in Fig. 2c, d are simulated lines that trace upper and lower polariton branches for the single-state coupling, showing the anti-crossing and all the parameters are maintained the same. The Rabi splitting is determined as about ${\hbar \Omega }_{{\rm{Rabi}}}=21.6$ meV. Using the experimental halfwidths for cooperative excitons (${\hbar \Gamma }_{{ex}}=10$ meV), and the cavity photon (${\hbar \Gamma }_{{\rm{Bm}}}=9$ meV), we can deduce the light-matter interaction potential $g=10.8$ meV via ${\hbar \Omega }_{{\rm{Rabi}}}=2\sqrt{{g}^{2}-1/4{({\hbar \Gamma }_{{\rm{ex}}}-{\hbar \Gamma }_{{\rm{Bm}}})}^{2}}$, which agrees with our calculation results ~10.5 meV^49^. This results in $g > \left|{\hbar \Gamma }_{{\rm{ex}}}+{\hbar \Gamma }_{{\rm{Bm}}}\right|$/2, a necessary condition for the formation of strongly coupled polariton states. In order to highlight the typical anti-crossing feature and conclusively demonstrate the formation of the new quasiparticle CEPs, the second-order derivative method is performed and presented in Fig. 2. However, in the control sample, the strong coupling can not take place and is shown in the supplementary information. It is crucial to recognize that quantum dots display distinct behaviors when they are linked with optical modes in comparison to perovskite single crystals. Generally, the Rabi splitting observed in perovskite crystal samples can reach up to several hundreds of meV^15,16^. On the other hand, individual quantum dots typically exhibit relatively low Rabi splitting values, typically falling within the µeV range^50–52^. This is primarily due to their smaller effective interaction cross-section for light-matter coupling, leading to a considerably weaker coupling strength. However, it’s worth noting that this interaction cross-section can be significantly enhanced when a cooperative exciton state is established in a thin film of perovskite quantum dots, resulting in a substantially increased coupling strength.

**Fig. 3:**
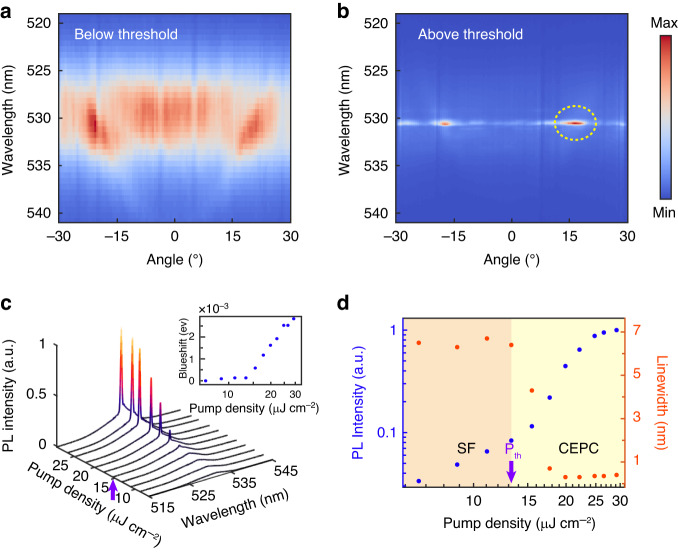
**Cooperative exciton-polariton condensation (CEPC)**. **a**, **b** Angle-resolved PL spectra for the QDs thin film on DBR below the pump threshold and twice the pump threshold. The yellow dashed circles highlight the condensation area. **c** PL spectra and the blueshift of the peak energy (inset) under various pump densities. **d** Evolution of the emission intensity and linewidth as a function of the pump density. The orange area represents the SF interval, and the yellow area represents the CEPC interval. The purple arrows in (**c**, **d**) represent the nonlinear threshold for the CEPC

The second phase transition from the SF to the so-called cooperative exciton- polariton condensation (CEPC) can be revealed when we increase the pump power above the nonlinear threshold as shown in Fig. 3c, d (Threshold is 15 μJ cm^−2^). Figure 3a, b display the angle-resolved PL spectra by pumping below the threshold and about twice the pump threshold. A significant intensity enhancement and linewidth reduction are observed at 17°. The angle of the condensation is built up exactly at the resonance position of the exciton and the Bragg modes. We attribute the exotic finite-momentum condensation to enhancing the density of states due to the coupling between the exciton and Bragg mode. Indeed, in our simulation, it is found that the total density of states of the coupled exciton-Bragg photon model achieves its maximum at wavelength 530 nm and near the anti-crossing position, which corresponds to the resonance position of the cooperative exciton and the first-order Bragg mode. Figure 3c shows the pump density dependence measurements, consistent with the onset of the condensation. Integrating over the range $\theta =16^\circ \sim 18^\circ$ and $\lambda =530$ nm ~ 532 nm, we can obtain the photon occupancy of CEPs at the condensation state ${I}_{p}$ ($\theta \sim$17°). At low pump densities, the photon occupancy increases linearly with the excitation, and then immediately after the pump threshold (the purple arrow shows), increases exponentially before becoming linear again. This sharp transition is accompanied by a decrease of the linewidth by about a factor of 30. Furthermore, as depicted in Fig. 3c, the observed blue shift measures ~2–3 meV, a value significantly smaller than half of the Rabi splitting. Consequently, it is evident that the lasing mode corresponds to the polariton mode rather than the Bragg mode. Therefore, the energy blueshift, as shown in the inset of Fig. 3c, along with the saturation and slight broadening of the above the 1.5 P_th_, can be attributed to the increased polariton- polariton interaction^53,54^.

**Fig. 4:**
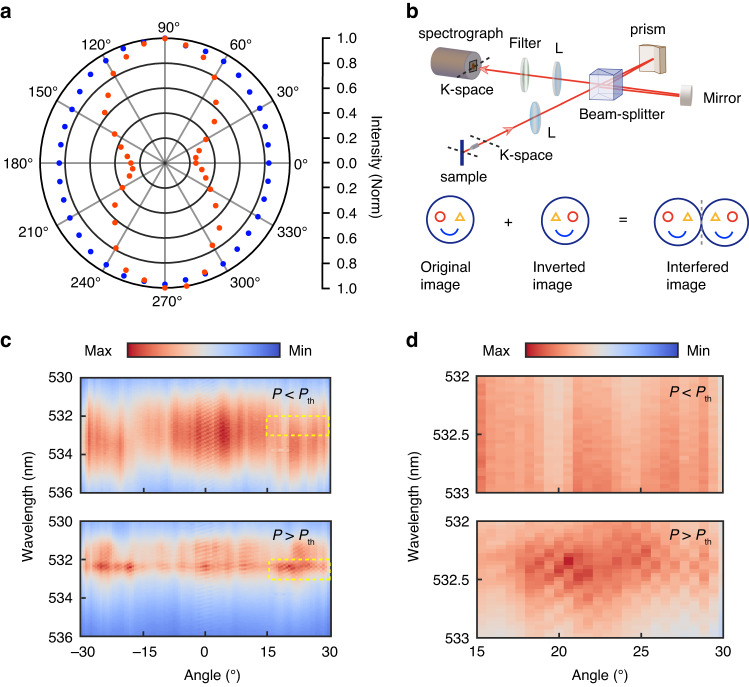
**Coherence and polarization properties of the cooperative exciton-polariton condensation**. **a** linearly polarization-resolved measurements below (blue dots) and above (red dots) P_th_ in the polar plot. The degrees of polarization are 9% and 83%, respectively. **b** Top, schematic of the Michelson interferometer setup. Bottom, illustration of the left and right symmetrically interfered images. **c** Typical interferogram for the angle-resolved PL spectra below and above the threshold. **d** Zoomed-in figures for the yellow dashed squares in c at the condensate state

Thereafter, we carry out polarization-resolved measurements to further study the optical properties of the emission for CEPC in k-space. The polarization of the pump laser is maintained the same. For the collection light path, we insert a linear polarizer for analyzing the emission polarization. Figure 4a shows the intensity integral measurement in different linear polarization directions (tune the direction of the polarizer of the collecting optical path) at the yellow dotted circle in Fig. 3b in the k-space. Further according to the data in Fig. 4a and the equation of $\frac{{I}_{\max }-{I}_{\min }}{{I}_{\max }+{I}_{\min }}$, we know that the degree of the polarization can be determined at about 83% for above the nonlinear threshold and appears to be completely depolarized below it. The high linear polarization of CEPC indicates the establishment of its coherence. Later, we investigate the coherence properties by using a Michelson interferometer. Here a right-angle prism and a retro-mirror are exploited to invert one of the images upside-down in according to the other one and is along the slit direction of the spectrometer which is displayed as the *x*-axis in Fig. 4c, d. We then apply a conjugated lens to convert the real space to the momentum space for checking the coherence under different angles. Figure 4c shows the typical interferograms below and above the threshold respectively with no time delay of the two beams. The interference fringes at the angle around 17° can only be observed when the pump power is above the threshold (illustrated by the yellow dashed squares in Fig. 4c). Otherwise, they are becoming invisible in our equipment test resolution limit. The yellow dashed squares are zoomed in and displayed in Fig. 4d. It is worth mentioning that at near ${k}_{\parallel }=0$ clear interference fringes can be seen all the time. It is because the images are reversed from one side to another, while the center of the image maintains the same. Therefore, the interference fringes at near ${k}_{\parallel }=0$ own to the self-coherence. Thus it can further confirm the establishment coherence at the two angles of 17° and −17°.

**Fig. 5:**
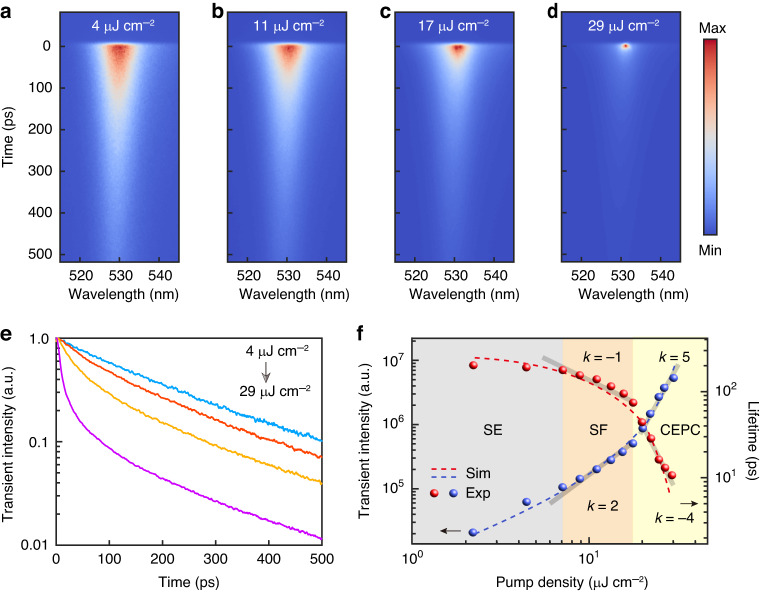
**Time-resolved spectra of the cooperative exciton-polariton condensation**. **a**–**d** Time-resolved PL spectra measured by a streak camera for different pump densities. (The excitation densities are 4 μJ cm^−2^, 11 μJ cm^−2^, 17 μJ cm^−2^, and 29 μJ cm^−2^, respectively). **e** Normalized transient PL curves following the pump densities, and the accordingly calculated radiation lifetimes are 190 ps, 137 ps, 73 ps, and 10 ps. **f** Pump density dependence of the transient peak intensity (blue dots) and the radiation lifetime (red dots), in log-log scale. The significant change in the slope of the lifetime and the transient intensity demonstrate the phase transition. The gray and orange areas are SE and SF intervals as discussed above, and the yellow area represents the cooperative exciton-polariton condensation interval. The transparent gray lines are the fitting lines for the trends *y* = x^k^. The corresponding k values in the CEPC interval are 5 for the transient peak and −4 for the lifetime. The red and the blue dashed lines are the calculation results overlaid with the experimental data

In addition to the observed intensity increase, the linewidth reduction and the energy blueshift, an increase in radiative decay rate is expected, hence, a shortening of the lifetime of the correlated exciton-polariton at condensation. Wavelength-resolved

lifetime measurements of the sample are taken by a Hamamatsu streak camera with a temporal resolution ∼1 ps. Figure 5a–d shows the time dynamics evolution under different excitation densities. A considerable reduction of the lifetime for the correlation exciton-polariton above the threshold is observed. The data from the lifetime maps are used to plot the lifetime dynamics curves for the various pump powers correspondingly as displayed in Fig. 5e. The radiation lifetime decreases from 190 ps to 10 ps when the pump density rises from 4 μJ cm^−2^ to 29 μJ cm^−2^. Pump density dependence of the transient peak intensity and the radiation lifetime are plotted on a log-log scale. The phase transition from the SF to the CEPC can be exhibited by the significant change in the slope from −1 to −4 for the lifetime and from 2 to 5 for the transient peak intensity shown in Fig. 5f. This is because, in conventional SF systems, the vacuum optical field plays a leading role in helping the establishment of exciton cooperation. However, in the CEPC region, this effect is further enhanced and accelerated by the stimulated radiation in the Bragg mode. Here, we conduct a theoretical simulation of the radiation process based on an open Dickie model coupled with a stimulated optical field (see supplementary information for more details). Through numerically solving the corresponding Lindblad master equation, we obtain the transient peak intensity and the lifetime of the radiation field (dotted lines in Fig. 5f), which agrees with our measurements in both the SF and CEPC regions.

## Discussion

In conclusion, we have demonstrated strong coupling between the cooperative excitons and Bragg photons in a perovskite QDs-based half cavity with a Rabi splitting of 21.6 meV. We further achieve the cooperative exciton-polariton condensation. The involved correlated excitons have proven to considerably enhancement of the coupling strength, which can be attributed to the cooperative effect inducing the synchronization of the random phases of the exciton to be aligned to form a giant dipole. Hence, it allows condensation to take place beyond what is possible at the individual QD level. The present demonstration of the new quasiparticle condensation enables new potential applications for developing ultra-narrow tunable lasers. Additionally, the possibility of controlling the condensation flow and hence exploiting it as the building blocks for various optoelectronic devices is another exciting field offered by such a perovskite QDs system.

## Materials and methods

### Preparation of CsPbBr_3_ QD thin film

CsPbBr_3_ QDs thin film are synthesized according to a literature procedure^55^. The highly concentrated resultant QDs solution in n-hexane is kept in a vibration-free environment at 10 °C for 3–5 days for low-temperature aging for further use. 1 cm × 1 cm Si substrates and DBR substrates are cleaned with acetone, ethanol, and deionized water, followed by O_2_ plasma treatment for 10 min. The QDs solution is spin-coated on the substrates at 1000 rpm for 10 s and then 3000 rpm for 30 s, left to dry for 5 min, and repeated 3 times.

### Experimental measurements

All optical experiments are performed in a high-vacuum closed-helium-cycle Dewar (Montana Instruments, cryostation-C2) at a temperature of 10 K. A femtosecond laser (450 nm) with a pulse duration of 300 fs and repetition of 200 kHz at normal incidence is employed as the pump source. PL signals were collected by a 50 × objective (numerical aperture: 0.5) in a confocal angle-resolved fluorescence detection system with a Michelson interferometer (Excipolar, Trion). The time-resolved PL measurements are obtained by a streak camera with a time resolution of ~1 ps (Hamamatsu, C10910).

### Theoretical calculations

The theoretical calculations are based on the coupled exciton-Bragg mode calculation. The Hamiltonian is described by the two-by-two matrix.

$$H({\bf{k}})=\left(\begin{array}{cc}{E}_{\text{ex}}-i{\varGamma }_{\text{ex}} & g\\ g & {E}_{\text{Bm}}({\bf{k}})-i{\varGamma }_{\text{Bm}}\end{array}\right)$$

Here ${E}_{\text{ex}}$ (${E}_{\text{Bm}}({\bf{k}})$) stands for the energy (dispersion) of the exciton (first Bragg mode), ${\varGamma }_{\text{ex}}$ (${\varGamma }_{\text{Bm}}$) represents the decay rate for the exciton (first Bragg mode) and $g$ is the coupling strength between the exciton and the first Bragg mode.

We can then extract the total (excitonic) density of states ${\rho }_{\text{tot}}$ (${\rho }_{\text{ex}}$) by calculating the imaginary part of the corresponding Green’s function,

$${\rho }_{\text{tot}}\left(E,{\bf{k}}\right)=-\frac{1}{\pi }\text{Im}\left[\text{Tr}G\left(E,{\bf{k}}\right)\right],{\rho }_{\text{ex}}(E,{\bf{k}})=-\frac{1}{\pi }\text{Im}\left[\eta G(E,{\bf{k}}){\eta }^{\text{T}}\right]$$

where $\eta =(\mathrm{1,0})$ is the vector correspond to the excitonic compnent and the Green’s function $G(E,{\bf{k}})={(E-H\left({\bf{k}}\right))}^{-1}\,$^56^.

In practice, we extract ${E}_{\text{ex}}$, ${\varGamma }_{\text{ex}}$, ${E}_{\text{Bm}}({\bf{k}})$, ${\varGamma }_{\text{Bm}}$ from the experimental data shown in Fig. 2c, d. The coupling strength $g$ is then fitted by comparing the calculated ${\rho }_{\text{ex}}(E,{\bf{k}})$ with the measured PL spectra.

### Supplementary information

Supplementary information
