# Supplementary material for: Observation of transition from superfluorescence to polariton condensation in CsPbBr3 quantum dots film
Source: Light Sci Appl. 2024 Jan 30;13:34. doi: 10.1038/s41377-024-01378-5 (PMC10828401; doi:10.1038/s41377-024-01378-5)
Supplement: Supplementary file 1 — Supplementary information [file 41377_2024_1378_MOESM1_ESM.docx]

**Supplementary Information for Observation of transition from superfluorescence to polariton condensation in CsPbBr_3_ quantum dots film**

Danqun Mao^1*^, Linqi Chen^2*^, Zheng Sun^1,3#^, Min Zhang^1^, Zhe-Yu Shi^1^, Yongsheng Hu^1^, Long Zhang^2^, Jian Wu^1,3,4,5,^ ,Hongxing, Dong^2#^, Wei Xie^1#^, Hongxing Xu^1,6^

*^1^State Key Laboratory of Precision Spectroscopy, East China Normal University, Shanghai, 200241, China*

*^2^Key Laboratory of Materials for High-Power Laser, Shanghai Institute of Optics and Fine Mechanics, Chinese Academy of Sciences, 201800 Shanghai, China*

*^3^Collaborative Innovation Center of Extreme Optics, Shanxi University, Taiyuan, Shanxi 030006, China*

*^4^Chongqing Key Laboratory of Precision Optics, Chongqing Institute of East China Normal University, Chongqing 401121, China*

*^5^CAS Center for Excellence in Ultra-intense Laser Science, Shanghai 201800, China*

*^6^School of Physics and Technology, Center for Nanoscience and Nanotechnology, Wuhan University, Wuhan 430072, China;*

***These authors contributed equally to this work**

^#^Email: [zsun@lps.ecnu.edu.cn](mailto:zsun@lps.ecnu.edu.cn);[hongxingd@siom.ac.cn](mailto:hongxingd@siom.ac.cn);[wxie@phy.ecnu.edu.cn](mailto:wxie@phy.ecnu.edu.cn);

**I:** **SEM image of CsPbBr_3_ quantum dot thin film**


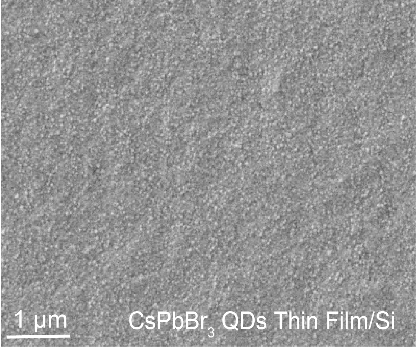


**Fig. S1.** SEM image of CsPbBr_3_ thin film consists of closely packing QDs with the typical thickness in a range from 200 nm to 500 nm. QDs are close packing.

**Ⅱ: Burnham–Chiao ring effect of CsPbBr_3_ QD thin film.**

**
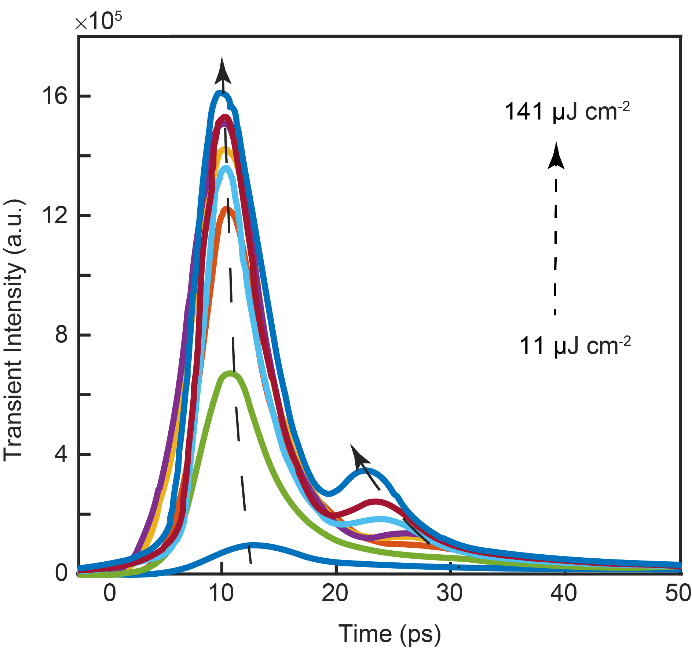
**

**Fig. S2.** Time-resolved photoluminescence spectra of CsPbBr_3_ quantum dot thin films on silicon under different pump densities measured at 10 K.

**Ⅲ: The time-correlated Coherence of Independent Exciton and Cooperative Excitons.**

We studied the temporal coherence using a Michelson interferometer. By tuning the relative distance between the two arms of the Michelson interferometer, we can introduce a time delay to study the temporal coherence.


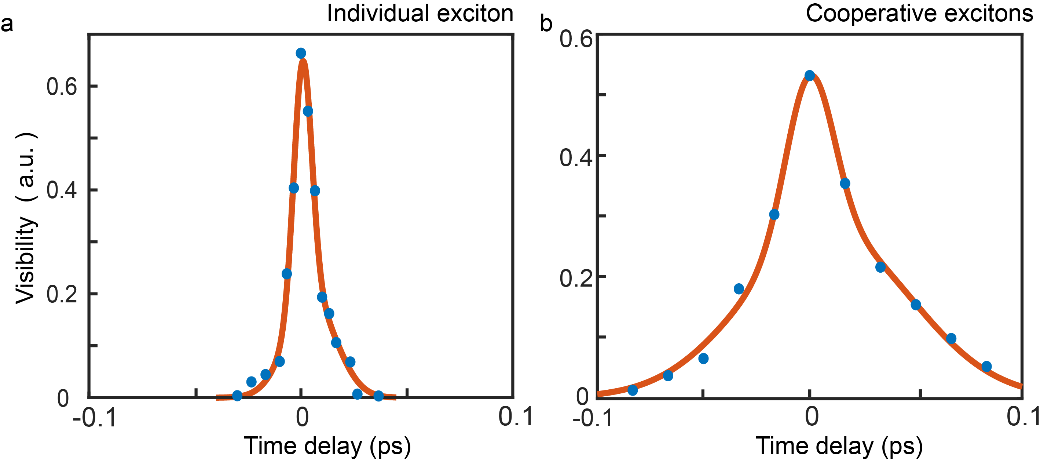


**Fig. S3. a, b,** Time-correlated coherence between independent excitons (unassembled monodispersed CsPbBr_3_ QDs) and cooperative excitons (CsPbBr_3_ QDs thin film) on the silicon. The coherence times are 40 fs and 200 fs, respectively, consistent with prior research findings on typical coherence times when the system transitions into the SF regime [1].

**Ⅳ: The time-correlated Coherence of CEPC**


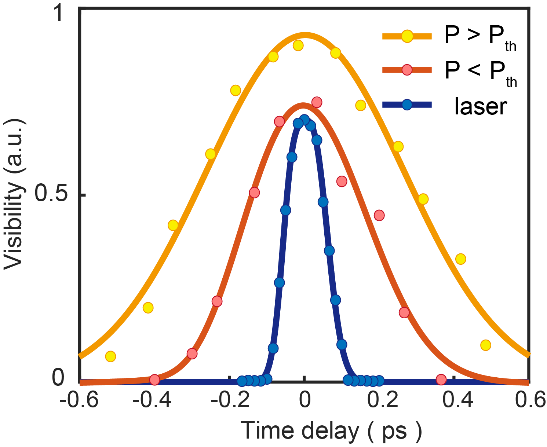


**Fig. S4.** Time-correlated coherence measurements followed up the same method as mentioned in section III were conducted both above and below the CEPC (Cooperative Exciton-Polariton Condensate) threshold, as well as for the pump lasers, using the complete sample structure, which comprises a thin film of quantum dots (QDs) on distributed Bragg reflectors (DBRs). The coherence times observed were 1.2 ps, 0.8 ps, and 0.2 ps, respectively. It is worth noting that the typical coherence time for the SF, as exemplified in Figure S3b for QDs on silicon, is 0.2 ps. Remarkably, the coherence time extends to 1.2 ps when transitioning from the SF phase to the CEPC phase. Additionally, the 0.8 ps coherence time suggests that the material state approaches the CEPC. Our experimental data indicates a linewidth of 1-2 meV in the CEPC (shown in Figure 3d) and the achieved 1.2 ps coherence time aligns with the quantum limit imposed by the uncertainty relation.

**Ⅴ: Coupling between the cooperative excitons and the first Bragg modes.**


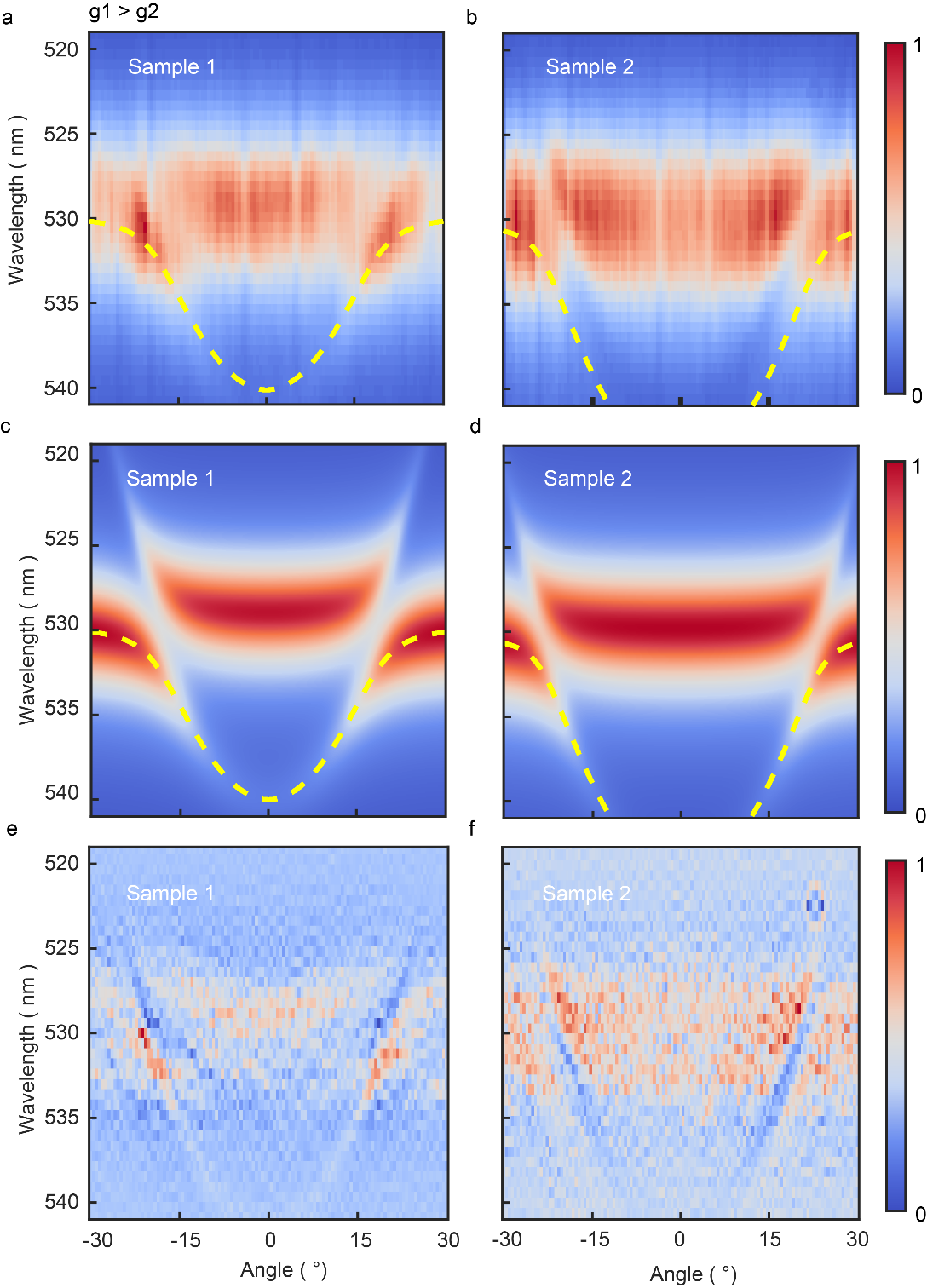


**Fig. S5. a, b,** Angle-resolved photoluminescence spectrum under the excitation density below the threshold for the different closely packed QD thin films. The anti-crossing dispersion feature and the opened energy gap are well resolved. **c, d,** the corresponding theoretical fitting for varying coupling strengths. The yellow dashed line represents the lower polariton branch. **e, f,** Second-order derivative processed data for Figure a-b respectively.

**Ⅵ: Coupling between individual exciton and the first-order Bragg mode.**


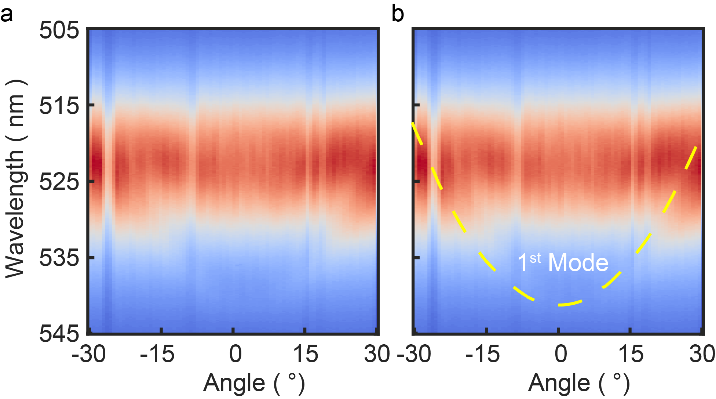


**Fig. S6. a,** The weak coupling of the individual quantum dot and first-order Bragg mode for the dispersed QDs on DBR. **b,** The yellow dashed line follows the dispersion of the first-order Bragg mode for guidance.

**Ⅶ:** **FDTD simulation for various quantum dot film thicknesses**

**
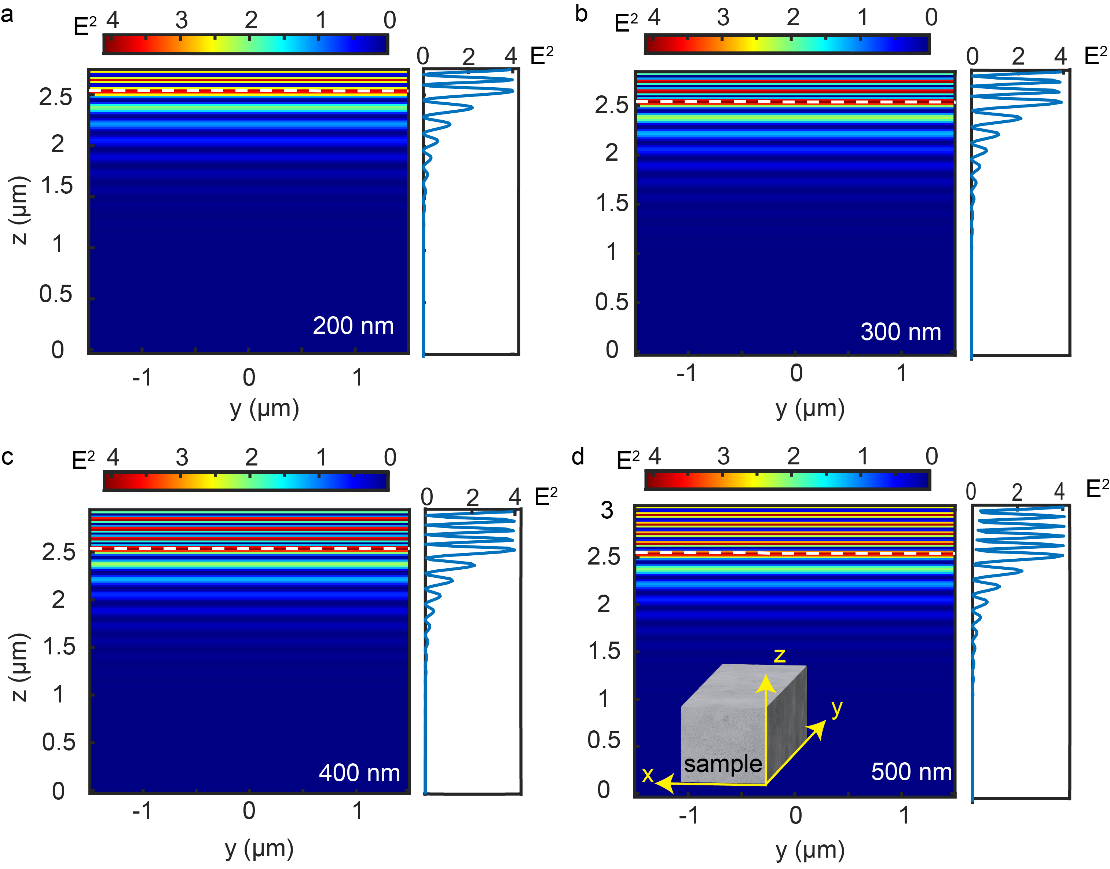
**

**Fig. S7. a, b, c, d,** illustrate FDTD simulations conducted on a quantum dot sample with thicknesses of 200 nm, 300 nm, 400 nm, and 500 nm. The white dashed line presents the boundary between the quantum dot thin film and the distributed Bragg reflector (DBR). Plane waves are directed from above, and the film's thickness is adjusted while tracking the electric field distribution within the ZY plane. Notably, the maximum intensity of the standing wave formed within the sample remains constant throughout the variations in sample thickness.

**Ⅷ: Theoretical Model:**

Conventional open system Dickie model usually discusses the decay of a few two-level atoms under the assumption that the system always couples to a vacuum light field. In this part, we shall generalize this model and assume that the system couples to a stimulated radiation field with average photon number $n_{p}$ instead. The many-particle Lindblad master equation should then be written as [2]

$\partial_{t}\rho=-\frac{\gamma}{2}\left( n_{p}+1 \right)\left\{ S^{+}S^{-},\rho\right\}-\frac{\gamma}{2}n_{p}\left\{ S^{-}S^{+},\rho\right\}+\gamma\left( n_{p}+1 \right)S^{-}{\rho S}^{+}+\gamma n_{p}S^{+}{\rho S}^{-}$ (1)

Here $\rho$ is the density matrix for N two-level (spin-$1/2$) degrees of freedom, $\gamma$ is the single-atom decay rate, and $S^{\pm}=\sum_{i=1}^{N} \sigma_{i}^{\pm}$ satisfies the commutation relation for the su(2) Lie algebra.

Now if we decompose the Hilbert space $H$ of these N spin ${-1}/2$ according to the irreducible representation of the su(2) Lie algebra of $S^{\pm}$, we have $H=H_{N/2}\oplus+\cdots$. Here $H_{N/2}$ is only irreducible representation space with dimension $N+1$ (or one may call it the spin-$N/2$ representation), which contains all the cooperative states of these N spins. A simple basis of $H_{N/2}$ would be

$\left. \left| \frac{N}{2} \right.,\frac{N}{2} \right\rangle,\left. \left| \frac{N}{2} \right.,\frac{N}{2}-1 \right\rangle,\cdots\left. \left| \frac{N}{2} \right.,-\frac{N}{2} \right\rangle$ (2)

And we have following relations for the su(2) operators

$S^{Z}\left. \left| N/2 \right.,m \right\rangle=m\left. \left| N/2 \right.,m \right\rangle, S^{\pm}\left. \left| N/2 \right.,m \right\rangle=\sqrt{\left( N/2\mp m \right)\left( N/2\pm m+1 \right)}\left. \left| N/2 \right.,m\pm1 \right\rangle$ (3)

where $S^{Z}=\frac{1}{2}\sum_{i} \sigma_{i}^{Z}$.

Because of the permutation symmetry of eq. (1), every irreducible representation spaces are decoupled. We may thus consider the evolution restricted in this subspace and write

$\rho=\sum_{m,m^{'}} p_{m,m^{'}}\left. \left| N/2 \right.,m \right\rangle\left\langle\left. \left| N/2 \right.,m^{'} \right| \right.$ (4)

with $\sum_{m,m} p_{m,m}=1$. The master eq. (2) can now be translated into

$\partial_{t}p_{m,m^{'}}=-\frac{\gamma}{2}\left( n_{p}+1 \right)\left[ \left( \frac{N}{2}+m \right)\left( \frac{N}{2}-m+1 \right)+\left( \frac{N}{2}+m^{'} \right)\left( \frac{N}{2}-m^{'}+1 \right) \right]p_{m,m^{'}}$

$+\gamma\left( n_{p}+1 \right)\sqrt{\left( \frac{N}{2}-m \right)\left( \frac{N}{2}+m+1 \right)}\sqrt{\left( \frac{N}{2}-m^{'} \right)\left( \frac{N}{2}+m^{'}+1 \right)}p_{m+1,m^{'}+1}$

$$-\frac{\gamma}{2}n_{p}\left[ \left( \frac{N}{2}-m \right)\left( \frac{N}{2}+m+1 \right)+\left( \frac{N}{2}-m^{'} \right)\left( \frac{N}{2}+m^{'}+1 \right) \right]p_{m,m^{'}}$$

$+\gamma n_{p}\sqrt{\left( \frac{N}{2}+m \right)\left( \frac{N}{2}-m+1 \right)}\sqrt{\left( \frac{N}{2}+m^{'} \right)\left( \frac{N}{2}-m^{'}+1 \right)}p_{m-1,m^{'}-1}$ (5)

Clearly, we see that the above equations are decoupled with respect to different $m-m^{'}$. If we are only interested in the average of the $S^{Z}$ operator, we have

$\left\langle S^{Z} \right\rangle=tr\left( \rho S^{Z} \right)=\sum_{m} {mp}_{m,m}$ (6)

Therefore, one may only consider the $m=m^{'}$ sector of eq. (5). The previous derivation ignores the phase of the light field and exciton. When superfluorescence occurs, the collective radiation is dominant at this phase and the absorption term can be ignored. In addition, consider the dephase ($\gamma_{\mathrm{dp}}$) of matter, we can reduce to following $N+1$ coupled equations,

$$\partial_{t}p_{m,m^{'}}=-\gamma\left( n_{p}+1 \right)\left( \frac{N}{2}+m \right)\left( \frac{N}{2}-m+1 \right)p_{m,m}$$

$-\gamma_{\mathrm{dp}}p_{m,m}+\gamma\left( n_{p}+1 \right)\left( \frac{N}{2}-m \right)\left( \frac{N}{2}+m+1 \right)p_{m+1,m+1}+\gamma_{\mathrm{dp}}p_{m+1,m+1}$ (7)

Note that the second line describes the process of the absorption of a background photon, and if we ignore it, the above equations are equivalent to eq. (12) in the supplementary information of Ref. [3].

The photon number $n_{p}$ changes due to the loss of the cavity and the radiation of the two-level atoms. The radiation rate I should be given by

$I=\gamma\left( n_{p}+1 \right)\sum_{m} \left( \frac{N}{2}+m \right)\left( \frac{N}{2}-m+1 \right)p_{m,m}$ (8)

The change rate of the photon number $n_{p}$ should then given by

$\partial_{t}n_{p}=I-{\gamma_{c}n}_{p}$ (9)

where ${t_{c}=1/\gamma}_{c}$ is the lifetime of the cavity photon.

To be noted, the corresponding parameters of the theoretical curves in Figure 5f are $\gamma=3.5\times{10}^{-4} {ps}^{-1}$, $\gamma_{c}=1.1 {ps}^{-1}$ and $\gamma_{\mathrm{dp}}=0.015 {ps}^{-1}$.

[1] Raino, G. et al. Superfluorescence from lead halide perovskite quantum dot superlattices. Nature 563, 671-675 (2018).

[2] M. O. Scully and M. S. Zubairy, Quantum optics (1999).

[3] C. Zhou, Y. Zhong, H. Dong, W. Zheng, J. Tan, Q. Jie, A. Pan, L. Zhang, and W. Xie, Nature Communications 11, 329 (2020).
